# Web GIS in practice III: creating a simple interactive map of England's Strategic Health Authorities using Google Maps API, Google Earth KML, and MSN Virtual Earth Map Control

**DOI:** 10.1186/1476-072X-4-22

**Published:** 2005-09-21

**Authors:** Maged N Kamel Boulos

**Affiliations:** 1 School for Health, University of Bath, Claverton Down, Bath BA2 7AY, UK

## Abstract

This eye-opener article aims at introducing the health GIS community to the emerging online consumer geoinformatics services from Google and Microsoft (MSN), and their potential utility in creating custom online interactive health maps. Using the programmable interfaces provided by Google and MSN, we created three interactive demonstrator maps of England's Strategic Health Authorities. These can be browsed online at  – Google Maps API (Application Programming Interface) version,  – Google Earth KML (Keyhole Markup Language) version, and  – MSN Virtual Earth Map Control version. Google and MSN's worldwide distribution of "free" geospatial tools, imagery, and maps is to be commended as a significant step towards the ultimate "wikification" of maps and GIS. A discussion is provided of these emerging online mapping trends, their expected future implications and development directions, and associated individual privacy, national security and copyrights issues. Although ESRI have announced their planned response to Google (and MSN), it remains to be seen how their envisaged plans will materialize and compare to the offerings from Google and MSN, and also how Google and MSN mapping tools will further evolve in the near future.

## Background

### The emergence of online consumer geoinformatics services

Mainstream Web search engines like Google  and MSN Search  have recently joined the geographic search bandwagon by releasing their own dedicated geographic interfaces, which run in standard Web browsers and also provide the general public with detailed satellite imagery/aerial photography map layers that were once only available to experts and select user communities.

### Google Maps and Google Earth

Google released Google Maps ( – a localized UK version is also available; see, for example, Paddington Station, London, W2 1RH (Satellite): ). Google also released Google Earth , a fat client, standalone 3D (three-dimensional) desktop application that offers anyone with Internet access a planet's worth of imagery and other geographic information, allowing users to virtually sightsee exotic locales like Paris, France, and Maui (Hawaii), Grand Canyon and Niagara Falls in the USA, as well as viewing points of interest such as local restaurants, hospitals, schools, and more. Google Earth uses KML (Keyhole Markup Language) to store data [1].

(It is noteworthy that NASA is also offering its own World Wind 3D application  that lets users zoom from satellite altitude into any place on Earth.)

Thanks to Google Maps API (Application Programming Interface – ), many third party applications and custom annotated maps have begun to appear [2]. Two good UK examples of such applications/custom annotated maps are the Health QOF (Quality and Outcomes Framework) Database map  and London July 2005 Terrorist Attacks map . (But because Google Maps has its roots in XML (eXtensible Markup Language), users were also able to produce their own custom annotated Google maps, e.g., based on their own GPS (Global Positioning System) locational data, and to even tie in images and video to create interactive multimedia maps, well before the API was publicly documented [3].)

Smugmug Maps  is another good example of a third-party Google Maps application in action. Smugmug, a photo hosting Web site, plots geocoded photographs to their actual locations on Google Maps (or Google Earth via a KML Google Earth feed: ), and allows location-based searching of photographs all over the world [4]. All smugmug RSS (Really Simple Syndication) feeds are now geo-enabled. If a photo has latitude, longitude, and altitude information (geographic metadata), it will show up in all feeds (see ).

### MSN Virtual Earth

Microsoft's response to Google Maps and Google Earth comes in the form of MSN Virtual Earth . A distinguishing feature of MSN Virtual Earth is its 'Locate Me' tool. Wired users can be located via their IP (Internet Protocol) address (this has been done for some time – see, for example, [5] and ). Wireless users can download a small application that does locating based on connection to a Wi-Fi access point. MSN Virtual Earth also features aerial oblique imagery (45 degree angle views or 'Eagle Eye Views') of major US metropolitan areas, provided by Pictometry International Corp. .

An MSN Virtual Earth Map Control/API (see  and ) allows users to create their own custom online maps, and add their own data to MSN Virtual Earth.

### Yahoo! Maps

Corresponding offerings from Yahoo! search engine have been modest by comparison, and include Yahoo! Maps  and an associated API . As at the time of writing, the Yahoo! Maps service does not offer any satellite imagery/aerial photography, but this might change in the near future. The latest traffic status/incidents, as well as Wi-Fi hotspots can be visualized on Yahoo! Maps. Gottipati [6] provides a useful comparison of Google Maps API and Yahoo! Maps API.

### Web browser toolbars and other developments

Dedicated Web browser toolbars and extensions have also started to appear, e.g., MutantMaps , a Mozilla Firefox toolbar that allows navigation between five popular mapping sites (Google Maps, MSN Virtual Earth, MultiMap.co.uk, TerraServer.com and 192.com) while preserving user's longitude, latitude and zoom levels, and gMapIt, another Mozilla Firefox extension that allows users to find directions from Google Maps based on publicly listed US phone numbers .

Along the same vein, Amazon.com is now also providing A9 Block View , an online Yellow Pages/map service that offers US maps with street-level photos.

### ESRI's response

Some commentators have recently wondered if users will soon eschew ArcGIS and ArcIMS (see  and ) in favour of using Google Maps API and MSN Virtual Earth API to quickly create Web map applications. ESRI's response to all of the recent online consumer geoinformatics services described in this article was to announce (in 2005) its new partnership with National Geographic , GlobeXplorer , and TeleAtlas , plus Geospatial One Stop (GOS – ) and a few other partners like MDA (MacDonald, Dettwiler and Associates Ltd. – ), to upgrade the National Geographic MapMachine , a map service/online atlas that provides global map coverage for an extensive set of Earth science themes. MapMachine was first launched in November 1999, and is powered by ESRI's ArcWeb Services . The planned upgrade aims at bringing satellite imagery, aerial photos, and street-level data to MapMachine users. Users will be able to access the service through a new viewer that is aimed at a mass audience, and appears to be ESRI's direct response to Google Earth and Microsoft Virtual Earth. However, one important difference from those services is that the ArcGIS back end will also allow users of the new service to accomplish much more sophisticated tasks, such as service area analysis. The next generation of MapMachine will also provide a link to GOS data and metadata to help users discover information about their area of interest or study. MapMachine will include capabilities for 3D globe services, allowing GIS users to "pull in" their own map services to overlay onto a globe. Also planned is the addition of ESRI's MapStudio , an ArcWeb Services application used by many daily newspapers to create maps for printing, to enable users to create customised maps [7].

## Methods

We wanted to experiment with the programmable interfaces provided by Google and MSN by mapping the headquarters locations of England's 28 Strategic Health Authorities (SHA), and associating these locations on the resultant maps with relevant online information about the corresponding SHAs from England's National Health Service (NHS) Web site .

### Geocoding

The programmable mapping interfaces provided by Google and MSN currently only accept longitude and latitude coordinates and do not provide their own geocoding services. Geocoding in our case was only based on the first part of SHA headquarters postcodes (e.g., PL12 for the South West Peninsula SHA, PL12 6LE), and was done using jibble.org list (see  and download at ). The worldKit geocoder , a free online worldwide city geocoder, also uses jibble.org list for UK postcode geocoding.

### Google Maps API version of our SHA map

Guided by Google Maps API online documentation  and Gottipati's online tutorial [6], we produced the Google Maps API version of our interactive SHA map. Google Maps API lets developers embed Google Maps in their own Web pages with JavaScript.

We had to first visit Google's sign-up page  to get a free API key for the Web site where our maps were to be published. API keys are site-specific. We included all SHA coordinates and Internet addresses (for accessing further information) in a separate XML file. An XSLT (eXtensible Stylesheet Language Transformation) stylesheet is used to display SHA information taken from this XML document.

**Figure 1:**
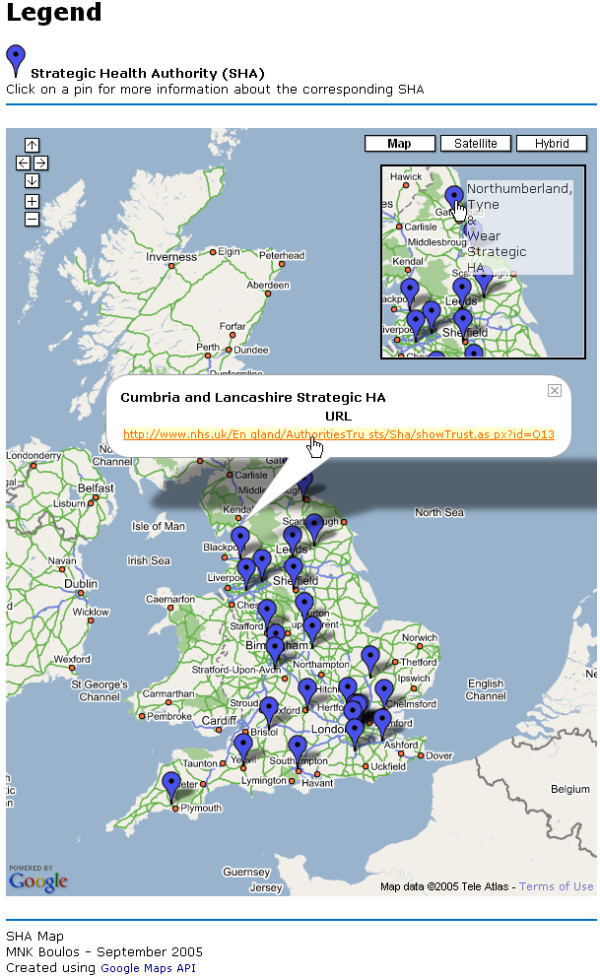
**Screenshot of Google Maps API version of England's SHA Locator. **Screenshot of our Google Maps API version of England's SHA Locator  showing the shadowed "info window" for Cumbria and Lancashire SHA, with a clickable external link to access further information about this SHA . The map features all the standard Google Maps controls for zooming, panning (also possible by dragging the map), and displaying/switching satellite and hybrid views. The inset shows an example of the tooltips that appear when the mouse hovers over the markers (or pins) on the map.

We used J. Shirley's GxMarker (see  and download at ) instead of Google's GMarker to have marker tooltips (see inset in Figure 1).

Readers wanting to further explore Google Maps API might be interested in Google Mapki Knowledge Base and list of developer tools . Google Mapki is a forum for sharing ideas, implementations, and help for the Google Maps API.

### Google Earth KML version of our SHA map

The development of the Google Earth KML version of our maps was again guided by Google's online documentation available at . KML is an XML-based language. A new MIME (Multipurpose Internet Mail Extensions) type (application/vnd.google-earth.kml+xml kml) must be added to the server hosting the KML feed file to help client Web browsers like Internet Explorer associate the file with the appropriate client application (Google Earth) rather than opening it as a plain XML file.

### MSN Virtual Earth Map Control version of our SHA map

Finally, we created a third version of our maps using MSN Virtual Earth Map Control (download control at ), and guided by Part 1 of Roodyn's excellent tutorial available from *Via Virtual Earth*, Virtual Earth developer resource centre [8].

It should be noted that not all possible features of the programmable mapping interfaces provided by Google and MSN have been explored or demonstrated in our exercise and its outputs (see 'Results' below). For example, it is also possible to add VML (Vector Markup Language) polyline overlays to maps created using Google Maps API. Also, Part 2 of Roodyn's tutorial describes additional controls and widgets that can be used with MSN Virtual Earth Map Control [9].

## Results

**Figure 2:**
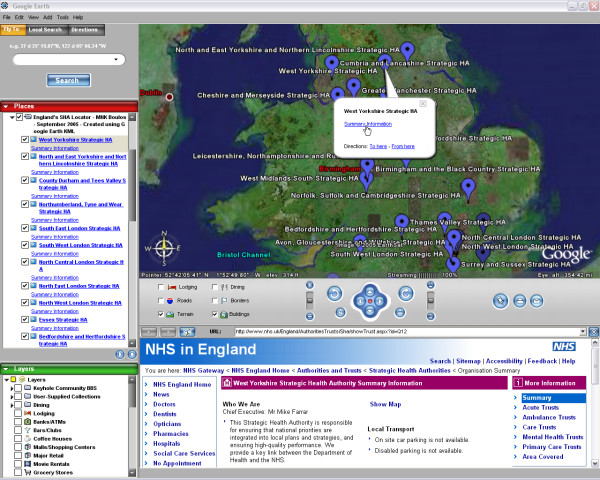
**Screenshot of Google Earth KML version of England's SHA Locator. **Screenshot of our Google Earth KML version of England's SHA Locator (see instructions at ) showing our KML SHA feed in Google Earth, with an "info window" for West Yorkshire SHA. "Info windows" allow users to access external Summary Information about the corresponding SHA ( in this example, displayed in the lower pane of Google Earth), as well as Google Earth-generated driving directions to or from the selected SHA. The KML feed featured in this screenshot is available at  and is intended to be opened by Google Earth desktop application, which can be downloaded at .

**Figure 3:**
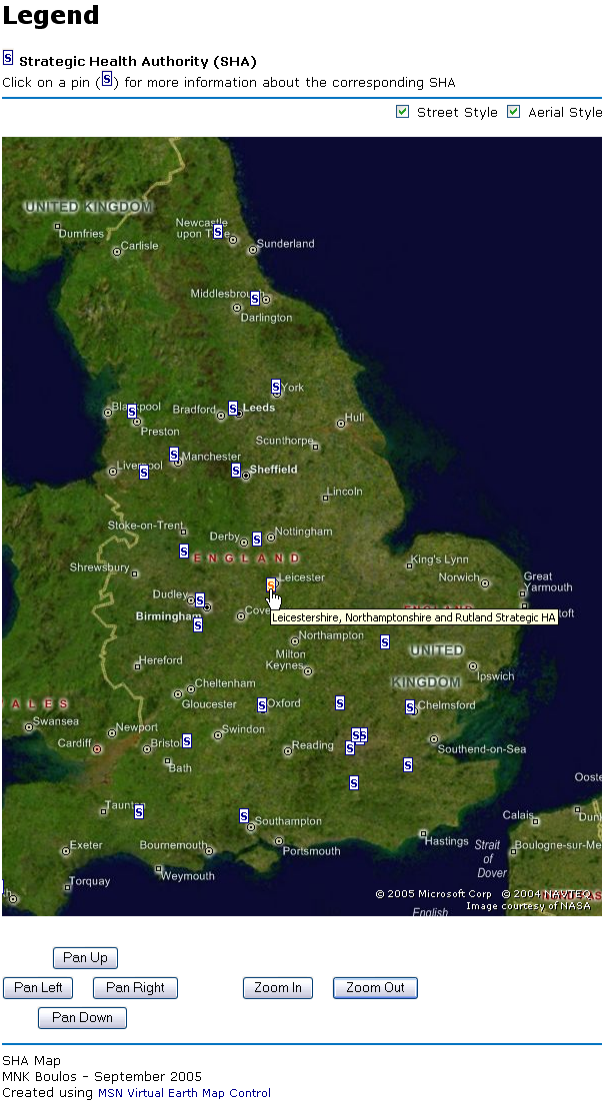
**Screenshot of MSN Virtual Earth Map Control version of England's SHA Locator. **Screenshot of our MSN Virtual Earth Map Control version of England's SHA Locator  in combined street and aerial (satellite) style modes. Like the main MSN Virtual Earth service, our custom map allows users to move the map around by dragging it, to zoom in and out with the mouse wheel, and to zoom in by double-clicking on a location. Clicking a Strategic Health Authority 'S' marker (or pin) on the map will display the corresponding Summary Information page from England's National Health Service (NHS) Web site (e.g.,  for Leicestershire, Northamptonshire and Rutland SHA).

The three interactive SHA map demonstrators we have created can be browsed online at  (Google Maps API version – Figure 1),  (Google Earth KML version – Figure 2), and  (MSN Virtual Earth Map Control version – Figure 3). The maps have been successfully tested in both Internet Explorer 6-SP2 and Mozilla Firefox 1.0.6 Web browsers.

## Discussion

### The geodata-rich society

ESRI president Jack Dangermond recently predicted that the supply of satellite and aerial imagery will increase by two folds in the next few years. Availability will also increase greatly, via Web portals and online GIS services. This is all part of what Dangermond describes as a "geodata-rich society" that will have access to more geospatial information of all kinds, including, in addition to imagery, GPS/location data, geo-demographic data, and data from real-time monitoring [10]. The Internet is already the 'foundation medium' to access, link and use all these data.

Satellite imagery and remote sensing are quickly entering the mainstream. Today, satellite imagery data are abundantly available from multiple sources, including companies such as Space Imaging , Orbimage , DigitalGlobe , GlobeXplorer , Spot Image , ImageSat International , and EarthSat ( – an MDA company), and are used in hundred of applications. But thanks to online consumer services like Terraserver , Google Earth, and Microsoft Virtual Earth (see 'Background' section above), satellite imagery has also been made familiar and accessible to millions of people.

### The wikification of GIS, maps and satellite imagery/aerial photography: imaging and geospatial information for the wide masses

There is no doubt the different online consumer geoinformatics services that have been presented in the 'Background' section of this article, including the different geographic search interfaces from major Web search engine providers, have significantly contributed (in record time) to raising the general public interest in geography and satellite imagery. As millions of people start "playing" with these new online "gadgets" or "toys" from Google and Microsoft, many of them will soon start thinking about becoming active participants, sharing information and collaborating online (notions that have been rightly associated with the Web for quite a long time), rather than just being satisfied with a passive information consumer/viewer role. (The reader should note that it has been estimated that about 800 million persons are online today worldwide [11].)

However, although Google Maps API (and similar API offerings from other providers) enables users to deeply customize the standard provider's interface (Google Maps), and to create their own custom annotated maps (custom applications based on Google Maps), such APIs remain difficult for the non-expert, average user to exploit. This author expects the technology to further evolve to enable the average Web user to share geospatial information, to customize, annotate and publish his/her own online maps and related Web applications, and to collaborate with other users/online communities within an online customizable and collaborative mapping environment, all without the need for any prior programming knowledge or expertise. (Such user-friendly applications that do not require end-users to have any programming expertise to use them can also be built using the existing APIs.)

The current 'wiki' concept is not far from this vision. A wiki (from Hawaiian *wiki*, to hurry, swift) is a collaborative Web site whose content can be edited by anyone who has access to it [12]. Perhaps the best example of a wiki in action today is 'Wikipedia – The Free Encyclopedia' (see the 'wiki' entry in Wikipedia at ). A related Web information sharing technology is the 'blog'. A blog (WeBLOG) is a Web site that contains dated entries in reverse chronological order (most recent first) about a particular topic. Functioning as an online journal, blogs can be written by one person or a group of contributors. Entries contain commentary and links to other Web sites, and images as well as a search facility may also be included ([13] – see the 'blog' entry in Wikipedia at ).

Wikis, and in particular Wikipedia, have grown very popular in recent months and years [14]. Wikis represent a promising principle that can significantly transform the Internet information age. Special conferences have been and are being organized to discuss this interesting Web phenomenon of wikis; for example, Wikimania 2005, the First International Wikimedia Conference, 4–8 August 2005, Frankfurt am Main, Germany , and the ACM (Association for Computing Machinery)-sponsored WikiSym 2005, the 2005 International Symposium on Wikis, 17–18 October 2005, San Diego, California, USA .

Along the same lines, it is not difficult to imagine the development in the very near future of 'geowikis', 'mapwikis', geo-enabled blogs, 'mapblogs' (imagine, for example, people with an Internet-connected, GPS-enabled mobile device wanting to blog their movements, and share their activity spaces and geo-referenced news with other online users for various purposes), and even geo-enabled, mappable Web/RSS feeds and map feeds (see the Smugmug KML photo feed example mentioned in the 'Background' section above). In fact some early geowiki examples have already found their way on the Web; see, for example, , , and also worldKit GeoWiki, a publicly editable map application  (a simple online demo of worldKit GeoWiki to which anyone can add their own data is available at ).

Another example is the Katrina Information Map , which was built using Google Maps [15]. Katrina Information Map was conceived for use by people affected by Hurricane Katrina (August 2005) and their relatives who have, or are trying to find, information about the status of specific locations affected by the storm and its aftermath. Users having information about the status of an area that is not yet on the map can easily contribute to the map by adding/appending their information to it. (Readers interested in Hurricane Katrina's online maps and imagery in general might also find the following two sites useful: , ,  and .)

The possibilities and potentials are endless. This is what this author calls the ultimate "wikification" of GIS, maps and satellite imagery/aerial photography. If the majestic Tate Museum in London is currently posting captions from its visitors next to its greatest works of art [16], why shouldn't online maps (even those from very reputable sources like the National Geographic Society) allow a similar approach?!

### Associated individual privacy, national security, data confidentiality, and copyrights/digital rights management issues

As geospatial technology progresses and becomes more readily available to the wide masses around the world who are connected to the Internet, the interrelated issues of GIS and map data confidentiality/individual privacy, and even national security start to surface, calling for further examination of, and research into these delicate aspects of Internet GIS and Web maps [5,17-22].

For example, in public health worldwide, any public identification of an individual's health status and residence, regardless of level of contagion or risk, is usually prohibited with very few exceptions, e.g., Megan's Law in the US, which allows the release of residential information on registered child sex offenders to the public by local government [17,23]. In fact, thanks to the latter law, we have a service like the Georgia Sex Offender Maps , which was built using Google Maps API. SARS (Severe Acute Respiratory Syndrome) mapping in Hong Kong in 2003 using disaggregate case data at individual building level in near real time was another noticeable exception to this well-established public health confidentiality rule, and also a unique and rare GIS opportunity that resulted in some very comprehensive public Internet mapping services [24].

It is noteworthy that Google Maps API terms and conditions  state, "There are some uses of the API that we just don't want to see. For instance, we do not want to see maps that identify the places to buy illegal drugs in a city, or any similar illegal activity. We also want to respect people's privacy, so the API should not be used to identify private information about private individuals."

On another level, following the September 2001 terrorist events in the US, many federal and local spatial databases, e.g., "critical infrastructure" spatial data, were assessed by their holding agencies as a potential liability to national security and withdrawn from the Internet or public dissemination. The current concern is to find an appropriate balance between public access to spatial information and protection of information considered a priority for national security [17,23].

But despite all these undeniable, legitimate and real concerns about Internet GIS and map data privacy and confidentiality, many of the doubts and misgivings that are raised concerning these aspects of Internet GIS seem to be ill founded, or at least exaggerated. Entchev [19] has wisely stated, "Let us not cripple the GIS system to meet some vague privacy perceptions".

Another thorny Internet GIS issue that needs to be addressed is that of data and map copyrights. Conner [14] has rightly described online maps as a copyright minefield. Copyrighted geo-data and maps are usually more difficult and expensive to acquire and use.

But as geo-data become more important in everything from blogs through mobile phones to finding lost people, free maps could make more and more of a difference [14]. However, someone needs to pay the bill for such "free" maps, and so finding sustainable commercial models for adoption by online geo-data and Web map providers is becoming of prime importance these days [25]. Examples of such commercial models include ad-sponsored map services, and low-cost, added-value paid services supporting the free service like Google Earth plus  and Google Earth Pro . Microsoft also provides an alternative ad-supported, but still free, "commercialized" version of their MSN Virtual Earth Map Control for commercial Web sites [26].

The Open Geospatial Consortium's (OGC) work on Geospatial Digital Rights Management (GeoDRM) is also poised to become an important enabler in the context of geo-data and map copyrights [27]. A great deal of work has already been done in the area of data ownership and rights management for the online e-book, video and music industries, with some mature working solutions already in existence from companies like Macrovision , Microsoft ( and ) and Adobe . Such developments are of interest to the geospatial community in that many geospatial data providers need to control or track who has access to their data and how the data are used. The lack of a GeoDRM capability has been identified as a major barrier to the broader adoption of Web-based geospatial technologies. The mission of OGC GeoDRM Working Group is to coordinate and mature the development and validation of work being done on digital rights management for the geospatial community [27].

## Conclusion

Google and MSN's worldwide distribution of "free" geospatial tools, imagery, maps and, eventually, in future versions of their products, analysis capabilities, is to be commended. Building on the powerful and universal visual language of geography, they succeeded in making their customizable multi-purpose maps and imagery of the world familiar and accessible to millions of ordinary Web users around the globe from outside the fields of specialized geosciences. Although ESRI have announced their planned response to Google (and MSN), it remains to be seen how their envisaged plans will materialize and compare to the offerings from Google and MSN, and also how Google and MSN mapping tools will further evolve in the near future.
